# Esophageal Squamous Cancer from 4NQO-Induced Mice Model: CNV Alterations

**DOI:** 10.3390/ijms232214304

**Published:** 2022-11-18

**Authors:** Zhiwei Liu, Ruibing Su, Anil Ahsan, Chencai Liu, Xiaoqi Liao, Dongping Tian, Min Su

**Affiliations:** Guangdong Provincial Key Laboratory of Infectious Diseases and Molecular Immunopathology, Department of Pathology, Institute of Clinical Pathology, Shantou University Medical College, Shantou 515031, China

**Keywords:** esophageal squamous cell carcinoma, 4NQO, whole-exome sequence, copy number variations, single-nucleotide variants, DNA damage

## Abstract

Squamous esophageal carcinoma is a common pathological type of esophageal carcinoma around the world. The prognosis of esophageal carcinoma is usually poor and diagnosed at late stages. Recently, research suggested that genomic instability occurred in esophageal cells during the development of esophageal squamous cell carcinoma (ESCC). Identifying prognostic and specific genomic characteristics, especially at the early hyperplasia stage, is critical. Mice were given 4-nitroquinoline 1-oxide (4NQO) with drinking water to induce esophageal cancer. The immortalized human esophageal epithelial cell line (NE2) was also treated with 4NQO. We performed histologic analyses, immunofluorescence, and immunohistochemical staining to detect DNA damage at different time points. Whole-exome sequencing was accomplished on the esophagus tissues at different pathological stages to detect single-nucleotide variants and copy number variation (CNV) in the genome. Our findings indicate that all mice were tumor-forming, and a series of changes from simple hyperplasia (ESSH) to intraepithelial neoplasia (IEN) to esophageal squamous cell carcinoma (ESCC) was seen at different times. The expression of γ-H2AX increased from ESSH to ESCC. In addition, mutations of the *Muc4* gene were detected throughout the pathological stages. Furthermore, CNV burden appeared in the esophageal tissues from the beginning of ESSH and accumulated more in cancer with the deepening of the lesions. This study demonstrates that mutations caused by the early appearance of DNA damage may appear in the early stage of malignant tissue before the emergence of atypia. The detection of CNV and mutations of the *Muc4* gene may be used as an ultra-early screening indicator for esophageal cancer.

## 1. Introduction

Esophageal squamous cell carcinoma (ESCC) is a major pathological type of esophageal cancer that accounts for 90% of all cases. It has a high incidence worldwide, including in Asia, East Africa, and South America [1,2]. The most prevalent etiologies associated with this condition are excessive drinking, smoking, inheritance, hot beverages, HPV infection, and achalasia [3]. Despite recent improvements in the prognosis, esophageal cancer remains the sixth most lethal malignancy globally due to the challenge of early diagnosis [4,5,6]. Therefore, finding early diagnostic indicators and pathogenesis is still an important issue in esophageal cancer research. Since it is challenging to obtain samples of early lesions or continuous lesions for research on the carcinogenesis of esophageal cancer, creating animal models is a crucial component of this field of study. In vitro carcinogenesis, chemically induced tumorigenesis, and genetically altered mice are three of the squamous esophageal carcinoma animal models that are now available. The frequency of use varies depending on the study goals [6,7,8,9]. The primary esophageal cancer model of C57BL/6 mice induced by 4-nitroquinoline 1-oxide (4NQO) is more appropriate to systematically study the pathological changes in the carcinogenesis process of esophageal cancer because it is possible to see intraepithelial neoplasia and invasive malignancy, which resemble human esophageal cancer [10].

4NQO is a water-soluble quinoline chemical carcinogen with a complex carcinogenic mechanism that produces all three stages of cancer: initiation, promotion, and progression [11]. It is activated in biological systems by the enzyme 4NQO reductase, and its active metabolite, 4NQO, is a biologically active carcinogen. The tongue and esophagus of mice contain a high concentration of 4NQO reductase, making them more susceptible to carcinogenesis when 4NQO is stimulated [12]. The production of reactive oxygen species during metabolism may have high cytotoxic and mutagenic potential, resulting in G mutation and DNA damage, including double-strand breaks [13,14,15,16]. 4NQO was used to induce oral squamous cell carcinoma in mice in the early stage. Tang [9] used it for the first time in establishing an esophageal squamous cell carcinoma model in 2004. This model demonstrated the specificity of the oral and esophageal epithelium and a high tumor formation rate. Therefore, 4NQO is likely to induce esophageal squamous cell carcinoma in mice by causing DNA damage and ROS production, which may play an important role in focusing on the correlation of DNA damage-related factors in primary esophageal cancer.

DNA damage is a prominent characteristic of tumors and is crucial to the genesis and growth of malignancies [17]. Accumulating ROS and other DNA-damaging chemicals in the tumor microenvironment leads to genomic instability of the body and causes disease [18,19]. Genomic instability includes microsatellite instability, increased rates of single base-pair mutation, and chromosomal instability. The most important form of instability in cancer genomes is chromosomal instability, mainly manifested as a replication or loss of genomic material, such as copy number variation (CNV) [20]. Therefore, it is also an important research direction to search for potential biomarkers for the early diagnosis of esophageal carcinoma from the direction of genomic instability. When employing this esophageal cancer model in earlier studies, the timing and characteristics of lesions at various stages of the cancer were rarely taken into account, and no exon sequencing studies with relevant stages were discovered. In our previous research, we observed the genomic characteristics of esophageal carcinoma in human esophageal carcinoma and cancer cell lines [21,22]. We found that DNA damage and inflammation played an important role in the carcinogenesis of esophageal carcinoma. Thus, in the current study, we used a 4NQO-induced primary ESCC model and whole-exon sequencing to examine the genetic characteristics of esophageal cancer in mice at various stages of simple hyperplasia, intraepithelial neoplasia, and esophageal cancer to understand the early carcinogenesis of squamous esophageal cancer.

## 2. Results

### 2.1. NQO Induction Esophageal Tumorigenesis in Mice

**Figure 1:**
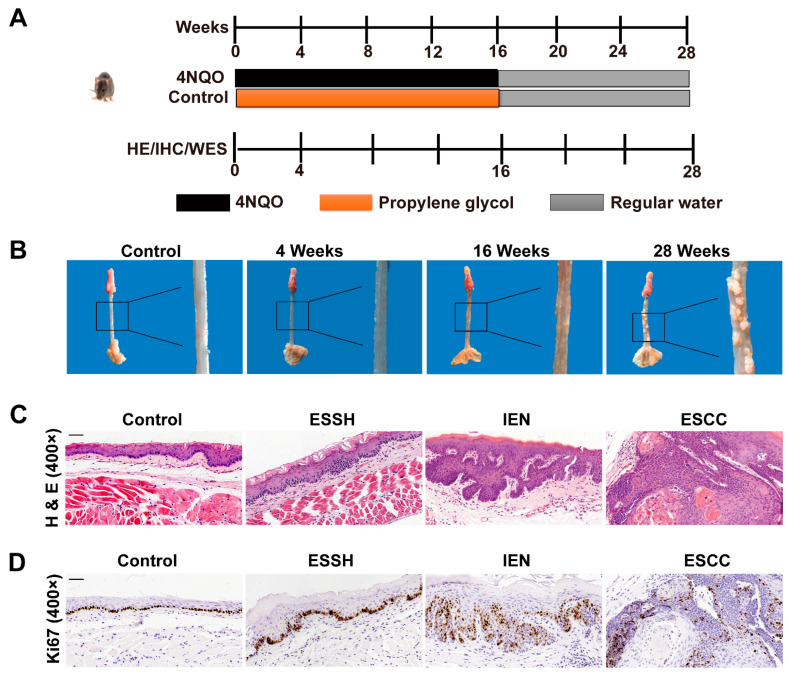
4NQO-induced neoplastic changes in esophagus tissues of mice at different time intervals. Control, normal; ESSH, simple hyperplasia; IEN, intraepithelial neoplasia; ESCC, esophageal squamous cell carcinoma. (**A**) Scheme for the treatment paradigm. C57BL/6 mice were randomized into two groups and fed with water or 4NQO (100 μg/mL) in drinking water for 16 weeks, after which all mice were given deionized water until week 28. Mice were sacrificed at different time points of 4NQO treatment at 0, 4, 8, 12, 16, 20, and 28 weeks to observe the different pathological stages of carcinogenesis in mice. The histopathological assessment, immunohistochemistry, and WES of esophageal tissues were performed after 4, 16, and 28 weeks. (**B**) Representative images of esophageal lesions for each stage. (**C**,**D**) Histological analysis and ki-67 staining of mice’s normal esophagus and ESCC esophagus for each stage. (Magnification, ×400; scale bar = 50 μm).

4NQO-induced esophageal cancer in mice is an established animal model for studying human diseases. In this study, we induced tumors in this manner. We observed obvious multifocal, precancerous, and cancerous lesions at different time points after carcinogen treatment for 28 weeks in all 4NQO-treated mice groups. We examined the rough esophagus tissue at the 4th week and gross cancerous esophagus lesion multiplicity (number of lesions per mouse) at the 16th week. After carcinogen withdrawal, the esophagus continued to deteriorate and eventually developed a 100% incidence of visible lesions at week 28. In contrast, no visible lesions were detected in the control mice not treated with 4NQO (Figure 1B).

We also did pathologic analyses on the esophagus sections from the 4NQO-treated mice. We found that continuous exposure to 4NQO carcinogen resulted in a gradual progression of carcinogenesis in mice, while esophageal tissues of the control showed normal epithelia. As shown in (Figure 1C), the mice’s esophagus sections contained different stages of esophagus carcinogenesis at different time points. At 4 weeks after 4NQO treatment, the mice developed lesions with simple hyperplasia, but no atypia of esophageal squamous epithelium was observed. Treatment with 4NQO up to 16 weeks was mostly found to be free of polarity in the epithelial cells and have nuclear pleomorphism, hyperchromatism, and increased or abnormal mitoses. Meanwhile, at 28 weeks, all 4NQO-treated mice exhibited typical pathological features of ESCC.

Moreover, we performed ki-67 staining to trace cell proliferation in the esophagus of the mice at different time points. A weak nuclear immuno-expression was observed in the mice of the control group. The mice exposed to 4NQO showed pre-tumorigenic features, such as increased cell proliferation, as indicated by increased nuclei and increased ki-67 staining of basal epithelial cells, which gradually increased with the prolonged induction of time (Figure 1D).

Taken together, these results show that 4NQO successfully induced esophageal tumorigenesis in mice, and the degree of these basic pathological changes is related to the time of stimulation and similar to the pathological changes of the human body.

### 2.2. DNA Damage Induced by 4NQO in Both Mice and NE2 Cell Line

**Figure 2:**
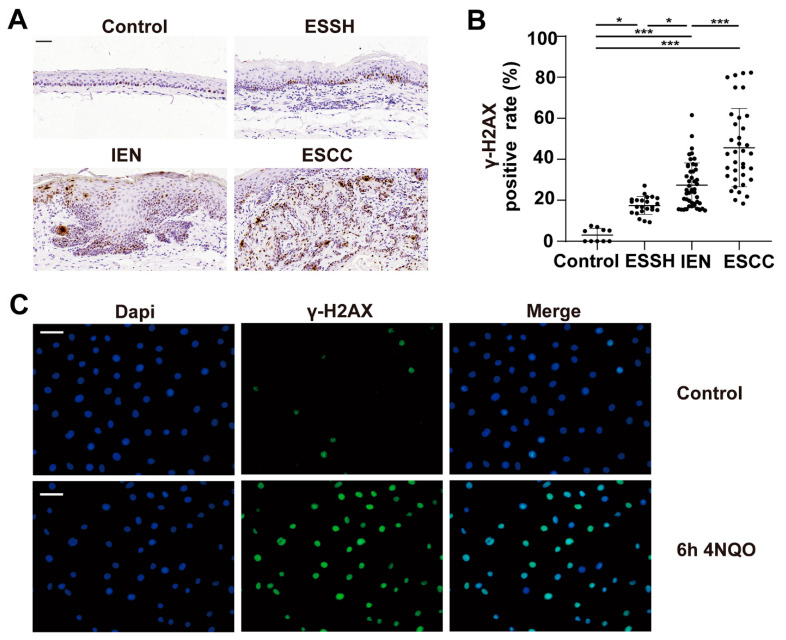
4NQO treatment enhances DNA damage in the esophagus of mice and the NE2 cell line. The mice were sacrificed, and the esophagus sections were fixed, embedded in paraffin, and sectioned. Then, the tissue sections were stained with γ-H2AX antibodies. Four to five representative areas of each esophagus section from each mouse per group were photographed and analyzed. (**A**) Immunohistochemical staining of γ-H2AX for each stage. (**B**) Statistical analysis of γ-H2AX positive cells for each stage. Differences are significant at * *p* < 0.05 ESSH, IEN vs control; *** *p* < 0.0001 ESCC vs. control, ESSH, IEN. The difference with *p* ≤ 0.05 was considered statistically significant. Magnification, ×400; scale bar = 50 μm. NE2 cell line treated with 4NQO for 6 h. γ-H2AX expression was visualized by immunofluorescence using primary specific antibodies and Alexa Fluor 488 conjugated secondary antibodies. Nuclei were stained with DAPI. (**C**) Immunofluorescence staining of γ-H2AX expression in NE2 cell line. Magnification, ×400; scale bar = 40 μm. Green fluorescence is γ-H2AX, blue fluorescence is Dapi.

DNA damage is involved in precancerous esophageal lesions and tumor initiation [23]. γ-H2AX phosphorylation is a sensitive sign of DNA double-strand breaks [24]. We determined DNA damage in the esophagus tissues of the mice at different time points through immunohistochemical investigations. Our results indicate that the immunoreactivity of γ-H2AX to ESSH, IEN, and ESCC in the 4NQO group was significantly higher than that in the control group (*p* < 0.001, Figure 2A,B).

To further support this notion, we next performed immunofluorescence analysis for γ-H2AX on a human esophageal immortalized epithelial cell line NE2 after the cells were treated with 100 µM 4NQO for 6 h, and the control group was treated with only 100 µM propanediol. Our results show that the expression of γ-H2AX increased in the 4NQO-treated cells than in the untreated cells (Figure 2C). These results suggest that 4NQO may play a role in DNA damage, and it caused significant DNA damage in the early hyperplasia stage of the mice’s esophageal epithelium before obvious atypia.

### 2.3. Mutated Genes in 4NQO-Induced ESCC Development

**Figure 3:**
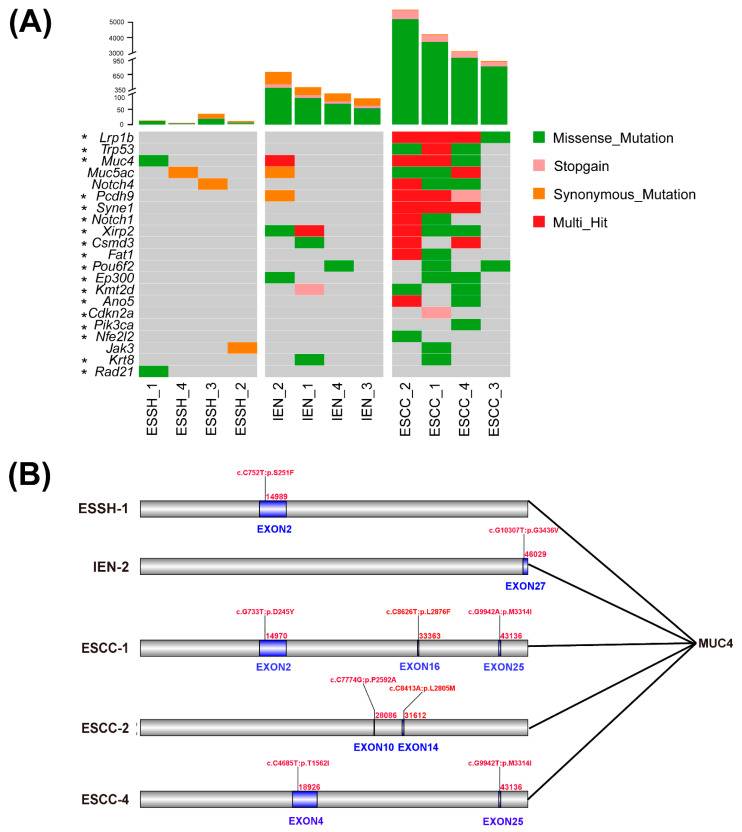
SNVs in different stages of ESCC mice model. (**A**) Top: total number of mutations (synonymous and non-synonymous mutations) in each stage of WES. Bottom: Significantly mutated genes detected by WES and * genes selected from the literature are ranked in the top panel. (**B**) Mutation sites of *Muc4* at various pathological stages.

We performed WES on 12 samples from 4NQO-treated mice to identify somatic alterations (SNVs and INDELs) at different stages of ESCC development (ESSH 4, IEN4, ESCC 4). We obtained different quantities of single-nucleotide variants (SNVs) and insertions/deletions (INDELs) in the ESSH, IEN, and ESCC groups. After alignment and data filtering, 366, 4230, and 39661 SNVs, were detected in the ESSH, IEN, and ESCC groups, respectively, and 32, 49, and 115 INDELs were recognized in the ESSH, IEN, and ESCC groups, respectively (Figure 3A, Supplementary Table S1). Next, we compared the nature of the somatic nucleotide variants at different stages of ESSH development. The mutation rates in the exon regions represent a mean increase in the ESSH, IEN, and ESCC samples (Supplementary Figure S1). C/G > T/A transitions were the predominant substitutions, which is consistent with our previous work and other published data, and they accounted for 30.58% (23090/75498) of all SNAs (Supplementary Figure S1) [21,22].

The above results are consistent with previous research on human esophageal cancer mutations [22,25,26,27,28,29], and we found that these genes (*Lrp1b*, *Trp53*, *Notch1*, *Pcdh9*, *Syne1*, *Xirp2*, *Csmd3*, and *Muc4*) showed mutations at high frequencies in our mice model. At the same time, we identified several of the most frequently mutated genes in this mouse model, including *Muc5ac*, *Notch4*, and *Jak3* (Figure 3A). Compared to the different stages of ESCC development, we observed that missense mutations of *Muc4* began to appear from the ESSH group, and more mutation sites were generated in the ESCC group (). Missense mutations of exon 2 appeared in both the simple hyperplasia group and the esophageal cancer group at similar sites (Figure 3B). On the other hand, missense mutations of *Muc5ac* and *Jak3* only appeared in the stage of ESCC. Furthermore, we identified that missense mutations of *Muc4* occurred at all stages, which might be an important molecular locus in the carcinogenesis of mouse esophageal cancer.

We checked the *Muc4* gene mutations of human esophageal squamous cell carcinoma on the COSMIC website. Muc4 missense mutations from 47 samples of human ESCC are shown in Supplementary Table S3. These mutations are also concentrated in exon 2, which are likely to be the sites found in mouse ESSH1 and ESCC1. Therefore, the mutations found in the mice are worth comparing with the human body. We also queried *Muc4* in our in-house data [22], and it was also observed that mutations of *Muc4* appeared in a patient (sample name: EC1110) during the ESSH, IEN, and ESCC stages (Supplementary Table S4).

### 2.4. Copy Number Variations

**Figure 4:**
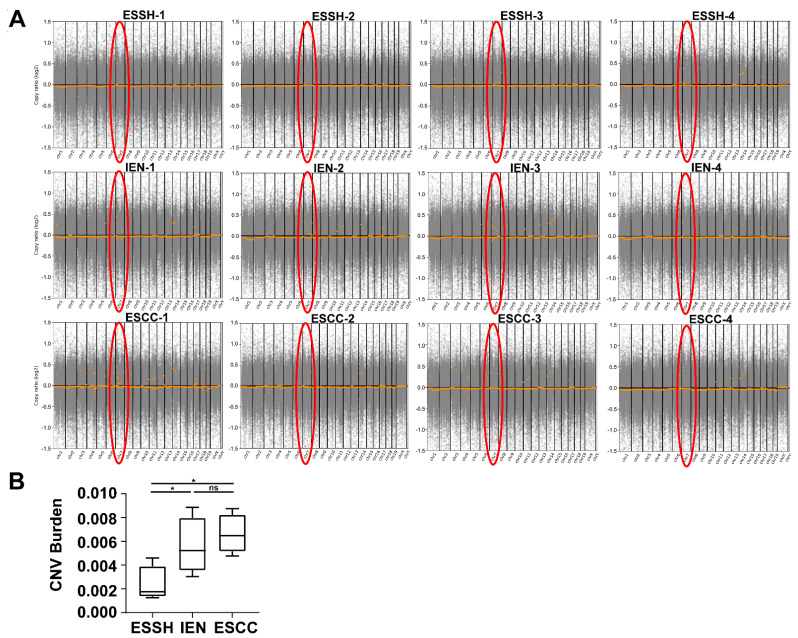
CNVs in different stages of ESCC mice model. (**A**) CNVs in chromosomes 1–19. The marked red circles indicate CNVs on chromosome 7 in each sample detected by WES. (**B**) CNV burden at different pathological stages (* *p* < 0.05, ESSH vs. IEN ESCC stage, ^ns^ *p* > 0.05 IEN vs. ESCC stage).

Copy number variations (CNVs) were analyzed to further explore 4NQO-related molecular events from 12 samples of 4NQO-treated mice (ESSH 4, IEN4, and ESCC 4). CNV amplifications started to appear at the hyperplasia stage and frequently accumulated in chromosome 7 at each stage (Figure 4A). We counted the number of CNVs at each stage, and there were 126 genes in ESSH, 201 genes in IEN, and 262 genes in ESCC (Supplementary Table S2). Moreover, we calculated the burden of CNVs at each stage of ESCC (Figure 4B). We detected that the CNV burden gradually increased from the simple hyperplasia stage to ESCC, with a statistical difference (*p* < 0.05) between IEN and ESCC. No significant CNVs were detected between IEN and ESCC. From the perspective of CNV burden, our data indicate that CNVs in the ESSH stage may be an important factor in the subsequent progression of esophageal cancer. In addition, compared to human tissues in our previous work, this model detected that the total number of CNVs was relatively small.

**Figure 5:**
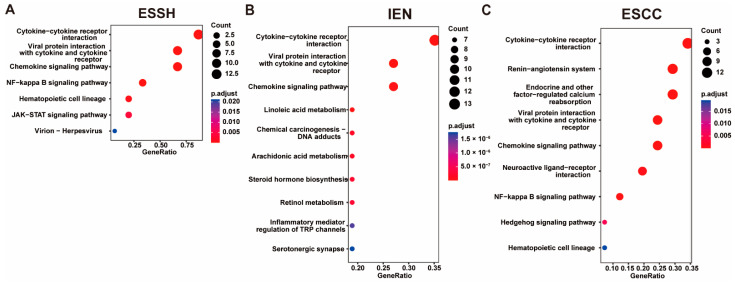
KEGG enrichment of CNV genes. ((**A**–**C**) TOP 10 KEGG pathway of ESSH, IEN, ESCC stage).

The CNV genes were enriched by KEGG pathways, and the results show that the pathways at each stage mainly acted as inflammation-related pathways (Figure 5). At the stage of ESSH, the CNV pathways mainly focused on the inflammatory activation pathways, such as the cytokine–cytokine receptor interaction, the chemokine-signaling pathway, and the NF-kappa B-signaling pathway. At the stage of IEN, not only the inflammatory pathways, such as ESSH, but also other pathways, such as the TRP channels, the Jak-stat-signaling pathway, and the neuroactive ligand–receptor interaction appeared. This indicates that the regulation of the pathway at IEN became complicated. The pathway of ESCC is similar to that of IEN. The cytokine–cytokine receptor interaction, the chemokine-signaling pathway, and the NF-kappa B-signaling pathway can be seen from the ESSH to the ESCC stage, with related genes such as *Gm10591*, *Ccl21b*, *Ccl19*, *Ccl21a*, and *Gm13304*. We found 122 CNV genes on chromosome 7 that were present in all three stages of ESCC, and these genes were mainly inflammatory chemokines. Interestingly, the *Nlrp9b* gene was also located on chromosome 7 in our mice model. CNVs of *Nlrp9b* were observed in ESSH-1 mice. In the meantime, the *Nlrp9b* copy number was amplified in IEN-3, ESCC-2, ESCC-3, and ESCC-4 mice.

## 3. Discussion

ESCC is among the most fatal malignancies worldwide, with increasing incidences and poor outcomes. 4NQO is a synthetic carcinogen and regarded as the best chemical to induce similar histological changes in animals to those of humans. Numerous studies have reported the 4NQO induction of oral and esophageal carcinoma in rat and mouse models [9]. However, the molecular mechanisms responsible for 4NQO-induced esophageal cancer have yet not been defined. Thus, we took this opportunity to analyze the genomic instability in 4NOQ-induced mice esophageal cancer. Our previous research indicated that the chronic inflammatory microenvironment in the early stage of esophageal cancer led to a large amount of DNA damage, and obvious CNV and mutation complexity were observed at the level of human tissues and cell lines through genomics [21,30,31]. Therefore, we continued to investigate DNA damage at different stages of cancer in the 4NQO mice model. In the current study, 4NQO produced high incidences of simple hyperplasia, intraepithelial neoplasia, and ESCC when observed at 28 weeks in the mice esophagus tissues (Figure 1). More importantly, local DNA damage significantly increased with the occurrence and development of cancer. Our further investigation found that somatic mutations and CNVs from simple hyperplasia to intraepithelial neoplasia to esophageal cancer gradually increased. These results are consistent with our previous studies on human esophageal cancer and cancer cell lines and provide additional evidence justifying the use of 4NQO in animal models of esophagus carcinogenesis.

The accumulation of excessive reactive oxygen species and reactive nitrogen species (RONS) due to carcinogen exposure contributes to esophagus carcinogenesis by causing oxidative modifications of DNA, proteins, and lipids [32]. Previous studies have demonstrated that ROS and RONS stimulated tissue repair and regeneration. However, if the body is unable to properly repair the damage that these chemicals cause to DNA damage, they may result in single DNA ruptures (SSBs), loss of bases, or disruption to genomic DNA replication and transcription. DNA double-strand breaks can lead to genomic instability [19,33]. A report indicated that 4NQO induces oxidative stress directly through ROS or indirectly through depleting GSH, thereby generating oxidative DNA damage [34]. In agreement with previous reports, the present study clearly indicates that the early-stage esophageal model is predicted to develop simple hyperplasia of esophageal cancer because the tumor rate is assumed to be 100%. This model differs from lesion specimens of simple hyperplasia tissue adjacent to carcinoma because it allows for a more precise prediction of the development of esophageal cancer at its most early stages. Interestingly, we found that the expression of γ-H2AX increased compared to the control (*p* < 0.05) in the simple hyperplasia stage of the esophagus of this model, indicating that the DNA damage at this stage began to accumulate. Meanwhile, the expression of γ-H2AX in NE2 cells increased after 4NQO stimulation for a short period, suggesting that it is possible that the pathological morphology of the cells has not yet been revealed atypia.

Genomic instability (mutations and CNV) is an important feature of tumors and plays an important role in the occurrence and development of tumors [17]. Muc4 is a member of the transmembrane mucin family and has many biological functions. It is involved in cell adhesion, and its EGF-like domain is involved in multiple signaling pathways by interacting with *ErbB2* and also affects apoptosis by activating the PI3K-Akt Pathway [35,36]. The complex relationship between *Muc4* and growth factor signaling is also reflected in the transcriptional regulation of *Muc4*, with interferon-γ, retinoic acid, and transforming growth factor β signaling pathways regulating *Muc4* expression in a partially interdependent manner [35]. In addition to being expressed in some normal epithelium, abnormal expression is also found in many cancers, such as breast and pancreatic cancer [37,38]. The query of ESCC Muc4 mutation in the COSMIC database suggested that *Muc4* may be an important mutated gene in ESCC. Mutations in *Muc4* have been found in various types of cancers, and studies on colon cancer and pancreatic cancer have reflected that *Muc4* mutation is associated with tumor immunity [39,40,41,42]. The current research on *Muc4* mutation reflects that it is associated with poor radiotherapy prognosis and prognosis of ESCC [29,43,44]. In our current study on mice, we also found *Muc4* mutations in mice ESCC. Interestingly, we found that Muc4 can first appear at the ESSH stage. Combined with the mutation data of human esophageal cancer in our in-house data, we found that *Muc4* mutation in human tissue samples also started to appear at the stage of ESSH, which suggests that *Muc4* may play an important role in the early screening of ESCC.

The expression of *Muc5ac* in esophageal adenocarcinoma may be more sensitive than that in ESCC [45,46]. However, it cannot be ruled out whether its expression is in ESCC. Therefore, *Muc5ac* mutation may be a new marker for ESCC. *Jak3* is an essential component of the *Jak* family, which is involved in cell growth, development, and the differentiation of various cells. It is also important for immune and hematopoietic cells [47]. Because of its critical role in immunity, deficiency of *Jak3* may lead to lymphocyte development and proliferation defects. Knockout mice may result in a phenotype dominated by defects in T-cell development and proliferation. However, *Jak3* has not been reported in many studies related to esophageal cancer. The somatic mutations of *Jak3* identified in ESCC may serve as an important supplement to the study of ESCC-related mutations.

CNV have been widely studied in recent years. Various previous studies have indicated that human genetic characteristics are common and influence the evolution of specific diseases. Recently, a genomic study related to esophageal cancer revealed that many characteristics of cancer have appeared due to the high frequency of CNV [25,31,48]. The development of CNV research has been used as a molecular diagnosis in various diseases [49,50,51]. In our model, CNV amplification genes were mainly enriched in inflammatory pathways. These results suggest that early inflammatory gene amplification may be an important factor in the progression of ESCC. Inflammation is closely related to tumors [17,52]. NF-kappa B pathways are crucial in tumors and inflammation, and relevant reports have been reported on esophageal cancer [53]. Our CNV enrichment results suggest that gene amplification in these inflammatory pathways may be of concern at an early stage. The inflammasome is a protein complex that plays a significant role in several diseases because it activates proptosis, which releases a number of inflammatory cytokines. Studies have indicated that proptosis plays a role in esophageal and gastrointestinal cancer [54,55]. Recently, the small intestinal epithelium of mice was shown to have an inflammasome known as *Nlrp9b*, which is capable of causing proptosis [56]. However, relatively little information is available regarding *Nlrp9b*’s involvement in esophageal cancer, particularly the changes to its CNV. Future research on its effects is still required.

Our previous findings on human esophageal cancer revealed links among mutations, intraepithelial neoplasia, and esophageal cancer stages [22]. Recent research using an organ-wide approach focused on the prostate showed that genome-wide copy number variation reveals unique clonal patterns within tumors and in surrounding benign tissue [57]. We also found this interesting phenomenon in our present model, where somatic mutations and CNV emerged from the simple hyperplasia stage to the esophageal cancer stage. Therefore, our findings suggest that it may be more valuable to carry out genomic screening of mutations and CNV in high-risk groups of esophageal cancer for prevention and ultra-early diagnosis.

## 4. Materials and Methods

### 4.1. Cell Line and Culture Conditions

NE2 cells (immortalized human esophageal epithelial cell line) were given by Prof. George Tsao from Hong Kong University. NE2 cells were grown in a defined keratinocyte-SFM medium and an Epilife medium supplemented with a defined keratinocyte-SFM growth supplement and an additional 1% FBS (Gibco, Grand Island, NY, USA). The cell cultures were maintained at 37 °C in a humidified atmosphere of 95% air and 5% CO_2_. The culture medium was renewed every three days. All experiments were performed with mycoplasma-free cells. The cell line was authenticated within the last 3 years using STR profiling.

### 4.2. Animals and Carcinogen Treatment

Six-week-old female C57BL/6 mice were purchased from Vital River Laboratory Animal Technology Co., Ltd. (Beijing, China). (NO. SCXK, 2019-0001). All the mice were raised in plastic cages according to sex, and the feeding density was no more than five per box in an air-conditioned room with a 12 h light/dark cycle and the temperature and relative humidity at 25 °C ± 2 °C and 50 ± 10%, respectively. All mice were fed a basal diet (Rodent Chow Product, KeAoXie Li feeds Co., Ltd., Beijing, China) and allowed free access to deionized water. All mice were reared in a specific pathogen-free (SPF) laboratory, and all the animal procedures and experiments conducted in this study were approved by the Animal Ethics Committee of Shantou University Medical College (Permit NO. SUMC2020-368). All efforts were made to minimize the suffering of the animals. We used 4-nitroquinoline 1-oxide (4NQO) from Sigma Aldrich (St. Louis, MO, USA) to induce esophageal lesions. The treatments were based partly on the work of Xiao-Han Tang et al. [9], with some modifications. Stock solution (1 mg/mL) was prepared weekly in propylene glycol, diluted in drinking water to a working concentration of 100 μg/mL, and stored at 4 °C. Drinking water containing 4NQO was freshly prepared every week using deionized water and administered to the mice in light-shielded water bottles. The design for the experimental studies is schematically shown in Figure 1A. After 1 week of quarantine, mice were randomly divided into 4NQO groups (n = 30), which were fed with water containing 4NQO (100 μg/mL) from the 1st to 16th week (shaded in black) and maintained with regular drinking water for a further 12 weeks (shaded in grey), and a control group (n = 5), which was fed with water containing propylene glycol from the 1st week to 16th week (shaded in orange), and then fed only normal water for a further 12 weeks (shaded in grey). Five mice were euthanatized with a 1% aqueous pentobarbital sodium solution at the 4th, 8th, 12th, 16th, 20th, and 28th weeks, respectively. The whole esophagus and stomach were opened longitudinally and cleaned with cold PBS. Esophagus lesions were detected under white light and carefully identified.

### 4.3. Histopathological Analysis

Tissues were fixed in freshly made 4% paraformaldehyde overnight at 4 °C, embedded in paraffin, and sectioned into 4 µm sections. A histopathologic assessment of esophagus sections was performed to examine the incidence of esophagus lesions. Whole-exon sequencing was performed on frozen esophagus tissues. Two certified pathologists conducted the histological determination of squamous neoplasia according to the 2019 WHO classification of tumors of the digestive system and the 8th edition of the Union for International Cancer Control—American Joint Committee on Cancer (UICC-AJCC) TNM staging system [58,59] at the Shantou University Medical College on the sectioned tissue samples. The esophagus sections were de-paraffinized, rehydrated, stained with H&E for histopathology, and observed under a light microscope. The ESCC lesions observed were separated into the following stages: Control: Well-oriented stratified epithelium consisting of the basal zone and superficial zone. Simple hyperplasia (ESSH): These abnormalities were confined to the lower third of the epithelium. Intraepithelial neoplasia (IEN): Defined as a loss of polarity in the epithelial cells, nuclear pleomorphism, hyperchromatic, and increased or abnormal mitoses. Esophageal squamous cell carcinoma (ESCC): Defined as a lesion with invasion into the sub-epithelial tissues.

### 4.4. Immunohistochemistry (IHC)

In addition to histologic evaluation, the esophagus sections were immunostained for the proliferation marker Ki-67 and oxidative DNA damage marker γ-H2AX. Immunohistochemistry (IHC) was performed to confirm the oxidative DNA damage on formalin-fixed paraffin-embedded (FFPE) esophagus sections using the Envision technique, Dako Real EnVision Detection System, and Peroxidase/DAB+ (Agilent Technologies, Santa Clara, CA, USA) according to the manufacturer’s protocol. Briefly, the FFPE sections were de-paraffinized with xylene, followed by rehydration in descending grades of ethanol. Endogenous peroxidase activities were blocked with 3% hydrogen peroxide for 15 min. Heat-mediated antigen retrieval was performed with a pressure cooker, in which the sections were immersed in citric acid buffer (10 mM, pH 6.0) at 125 °C for 3 min, followed by cooling at room temperature and washing with phosphate-buffered saline (PBS). To block nonspecific staining, we incubated the sections with 10% normal goat serum (ZSGB-BIO, ZLI-9022, Beijing, China). Next, the slides were incubated at 4 °C overnight with primary antibodies specific for γ-H2AX (dilution 1:100, #9718, Cell Signaling Technology, Danvers, MA, USA) and Ki-67 (dilution 1:500, #12202, Cell Signaling Technology, Danvers, MA, USA). After washing with PBS, the FFPE sections were incubated with secondary antibodies (Max Vision HRP Rabbit kit, MXB Biotechnologies, Kit-5004, Fuzhou, China) at 37 °C for 30 min. Diaminobenzidine (DAB) was applied for 5 min for visualization (DAB Kit, MXB Biotechnologies, DAB-0031, Fuzhou, China). Subsequently, the FFPE sections were washed with water, counterstained with Harris hematoxylin, followed by dehydration with ethanol, cleared with xylene, and mounted on cover glasses. Immunohistochemical staining was observed blindly by two independent pathologists. Images were captured using a Leica IM50 microscope at ×400 magnification, and five different fields for each index were selected in each sample. For the quantification of γ-H2AX expression, the numbers of positive nuclei and total squamous epithelial cells in the images were counted, and the corresponding immunostaining positive rates were computed as positive nuclei/total nuclei ×100%.

### 4.5. Immunofluorescence Staining

Immunofluorescence staining was accomplished against phospho-H2AX to examine the oxidative DNA damage in the NE2 cell line. Briefly, cells were harvested when they reached 80% confluence. Then, immortalized human esophageal epithelial cell lines NE2 (1 × 10^5^) were inoculated onto a coverslip in a 24-well plate and incubated for 24 h at 37 °C. Following incubation, the cells were treated with 100 µM 4NQO, and the control group was treated with only 100 µM propanediol for 6 h at 37 °C. Subsequently, the cells were fixed with 4% paraformaldehyde at room temperature for 30 min, permeabilized with 0.5% Triton X-100 at room temperature for 30 min, and washed with PBS three times after each treatment. Primary antibodies of phospho-H2AX (1:300 dilution) were applied to the slide and incubated in a humidified box at 4 °C overnight. A secondary goat anti-rabbit antibody conjugated with Alexa Fluor^®^ 488 was applied after PBS washing three times for 5 min each, put in a black wet box with shading, and then incubated in a humidified chamber at 37 °C for 30 min. After incubation, the slide was washed with PBS and mounted using antifade mounting with DAPI. The cells were then visualized under a fluorescence microscope (Zeiss, Jena, Germany) utilizing a 488 nm excitation. Fluorescence images were captured by ZEN imaging software (ZEN 2011; Carl Zeiss, Jena, Germany). Γ-H2AX foci numbers were measured in at least 200 cells per treatment group.

### 4.6. Isolation of Genomic DNA

The whole esophagus was taken at the 0th, 4th, 16th, and 28th weeks. H&E staining was performed on the first and middle portions of the frozen esophagus sections to confirm the pathological changes. DNA was isolated from the remaining portion of tissues by using the All Prep DNA/RNA Kit (QIAGEN; Valencia, CA, USA) following the manufacturer’s instructions. The normal esophagus served as a DNA reference for germline mutations. Sample integrity and yield were assessed by the Qubit^®^ Fluorometer (Thermo Fisher Scientific, Waltham, MA, USA). Purified DNA samples were stored at −20 °C.

### 4.7. Whole-Exome Sequencing (WES)

A total of 0.6 μg of genomic DNA was subjected to WES performed at Beijing Novogene Bioinformatics Technology Co., Ltd. (Beijing, China). A paired-end DNA library was created using the Agilent SureSelect^®^XT method with on-bead modifications. Genomic DNA was randomly fragmented to 180–280 BP with Covaris cracker (Covaris, Woburn, MA, USA). Fragmented DNAs were tested for size distribution and concentration using an Agilent Bioanalyzer 2100 and Nanodrop. After PCR amplification and quality control, the Agilent SureSelect Mouse All Exon V1 kit was used to enrich, hybridize, and capture the obtained fragments following the manufacturer’s procedures. DNA sequences (50 Mb) of 221,784 exons of 24,306 genes were captured. Whole-exome sequencing was performed on the Illumina HiSeq4000 platform using the PE150 sequencing strategy. The total exon region of mouse esophageal tissue was sequenced at a sequencing depth of 100×.

### 4.8. Bioinformatics Data Analysis

First, data quality control was performed, and all downstream bioinformatics analyses were based on high-quality clean data, in which reads containing an adapter, reads containing poly-N, and low-quality reads were removed. Following sequencing on the HiSeq platform, the primary data were converted into a FASTQ format and processed to retrieve high-quality paired-end reads, which were aligned to reference the genome sequence reference genome (UCSC mm10) using the Burrows–Wheeler Alignment tool [60] and Samblaster to obtain the initial comparison results in BAM format. The BAM files were marked repeatedly with Samblaster to obtain the final comparison results in BAM format [61]. High-quality alignment was essential to guarantee the variant calling accuracy (greater than 0) to identify single-nucleotide polymorphisms (SNPs) and insertions–deletions (INDELs). The analysis-ready Binary Alignment/Map (BAM) alignment results were produced after being processed using SAMtools. We mainly used muTect software [62] to find somatic SNV sites and Strelka to detect the somatic INDEL information. Copy number variations (CNVs) were detected using CNVKIT software. CNV Burden: The total genomic regions spanned by continuous somatic CNV segments were summed up, and the final CNV percentage was calculated based on the size of the autosomal mouse genome [63,64].

### 4.9. Statistical Analyses

Statistical significance in the experiments was assessed using GraphPad Prism, version 8.02 (La Jolla, CA, USA), and statistical software R (V 4.0.3). All data were collected and analyzed blindly and presented as the mean ± SD. Statistical evaluations of the post-hoc multiple group comparisons were conducted using one-way and two-way analysis of variance (ANOVA), followed by Tukey contrasts. A non-parametric test was used to compare the CNV burden. A *p* value ≤ 0.05 was considered statistically significant; NS, non-significant. Mutations of the human ESCC were checked on COSMIC (https://cancer.sanger.ac.uk/cosmic, accessed on 1 November 2022). KEGG enrichment was carried out using software R (clusterProfiler package), showing the top 10 pathways, *p*.adj < 0.05.

## 5. Conclusions

Our current study demonstrates that 4NQO-induced pre-malignant and malignant lesions in the esophagus of mice not only pathologically and morphologically mimicked human esophageal cancer but also shared similar genetic alterations (somatic mutations and CNVs). Furthermore, our data indicate that DNA damage may have occurred at the super early stage of carcinogenesis, and eventually, accumulated qualitative changes led to intraepithelial neoplasia and cancer. The data can be used to model the key genetic events in esophageal tumorigenesis as potential biomarkers for early detection and provide new insights into the architecture of ESCC progression. However, there are some limitations to this study. The 4NQO-induced mice ESCC model can simulate the development process of human ESCC. Some of our results are similar to the previous findings on human esophageal samples, but some of our results are novel, and care should be taken when applying these results to humans. We also did not manipulate other relevant mechanisms. Further studies are warranted to clarify these issues and to make this animal model more useful for research.

## Data Availability

All datasets used and/or analyzed during the current study are available from the public database NGDC (https://ngdc.cncb.ac.cn/, upload on 24 October 2022). The project number is PRJCA012714.

## Supplementary Materials

The following supporting information can be downloaded at: https://www.mdpi.com/article/10.3390/ijms232214304/s1.
