# Supplementary material for: Esophageal Squamous Cancer from 4NQO-Induced Mice Model: CNV Alterations
Source: Int J Mol Sci. 2022 Nov 18;23(22):14304. doi: 10.3390/ijms232214304 (PMC9698903; doi:10.3390/ijms232214304)
Supplement: Supplementary file 1 [file ijms-23-14304-s001.zip › Supplementary Figure.pdf]

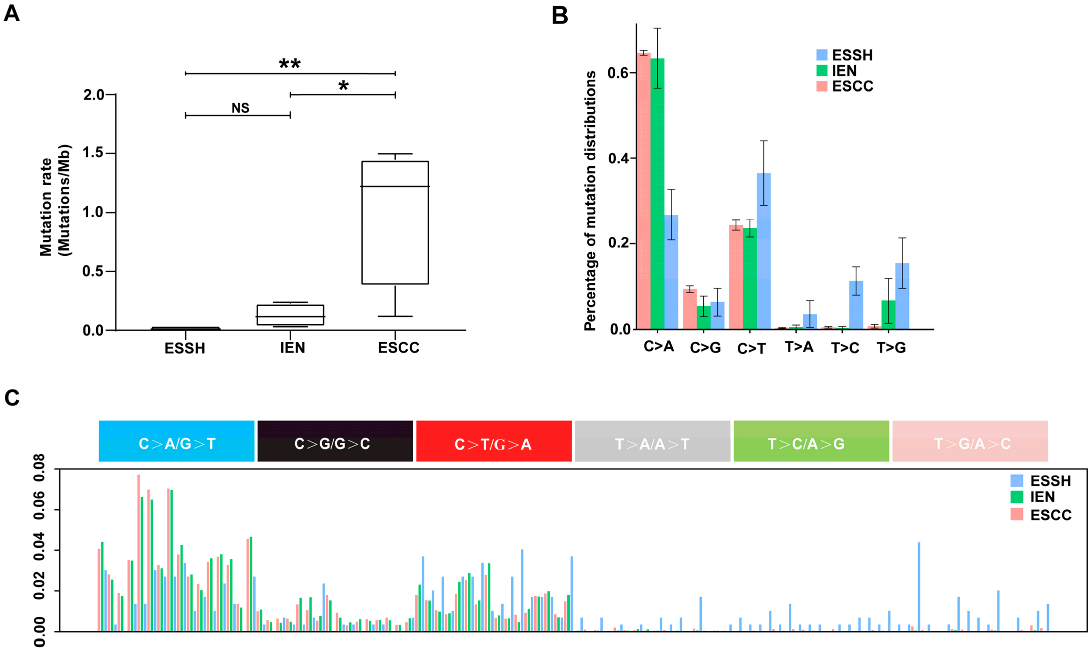

Figure S1. Characteristics of somatic mutations in different stages of ESCC (**A**). Comparison of mutation rate among groups, ESCC VS ESSH, *, $p < 0.01$, ESCC VS IEN, $p < 0.05$; (**B**,**C**). The mutation characteristics of each group, blue is ESSH , green is IEN, pink is ESCC）
